# Thermal imaging of local skin temperature as part of quality and safety assessment of injectable drugs

**DOI:** 10.1016/j.heliyon.2023.e23417

**Published:** 2023-12-15

**Authors:** Aleksandr Urakov, Natalya Urakova, Aleksandr Samorodov, Petr Shabanov, Ilnur Yagudin, Anastasia Stolyarenko, Darya Suntsova, Nikita Muhutdinov

**Affiliations:** a Department of General and Clinical Pharmacology, Izhevsk State Medical University, Izhevsk, Russia; b Department of Inventions and Patents, Institute of Thermology, Izhevsk, Russia; c Department of Pharmacology, Bashkir State Medical University, Ufa, Russia; d Department of Neuropharmacology, Institute of Experimental Medicine, Saint Petersburg, Russia

**Keywords:** Temperature, Infrared imaging, Drug, Injection, Inflammation, Safety

## Abstract

Injection of high-quality drugs can occasionally cause unexpected and unexplained local complications. As the current standard for drug quality control does not include an assessment of the local irritation effects of drugs, this effect may cause postinjection complications. Simultaneously, local irritation effects of the drugs can be assessed based on local tissue inflammation. The dynamics of local temperature can assess inflammation. Infrared monitoring of local skin temperature dynamics at subcutaneous, intramuscular, and intravenous injection sites of drugs under experimental and clinical conditions can improve their quality and safety. Therefore, there is a need to include dynamic thermography in the standard of biological evaluation of the quality and safety of drugs in the dosage form “solution for injections.” This eliminates the local irritation and necrotizing activity of drugs and minimizes the development of local pain syndrome, aseptic inflammation, necrosis, and abscess.

## 1 Value of the data

1. Why are these data useful?

These data provide a new perspective on pharmacological assessment of local irritant action, as well as on forensic examination of the local safety of drug solutions during injection.

2. Who can benefit from these data?

Medical thermal imaging manufacturers, drug manufacturers, medical professionals who use injections, and forensic examiners can benefit from the data presented in this article.

3. How can these data be used/reused for further insights or development of experiments?

These data can be used to develop new types of thermal imaging devices and medical injection rooms and new technologies for monitoring drug safety in intensive care and anesthesiology departments. Furthermore, these data can be used to modernize the pharmacopoeia and its article “Quality Control of Drugs.”

## 2 Introduction

Injections lead enteral and parenteral drug administration in treating various diseases. ’‘’Intravenous (IV) administration of drugs is common. Intramuscular injections are the second most common injection [[1], [2], [3], [4], [5], [6]]. However, subcutaneous injections are rarely used [7]. While intravenous injection technology is the most difficult, expensive, and dangerous to patient health, subcutaneous injection technology is the easiest, cheapest, and least dangerous [8,9]. Emergency departments, anesthesia, and intensive care units (where most injections are administered) administer drugs mainly via intravenous injections. In these hospital wards, intravenous drug injections are not administered with conventional syringes and thin injection needles, but with complex systems with thicker and longer intravascular catheters. These catheters stay in blood vessels (typically superficial veins) for days [10]. Therefore, intravenous drug injections are complicated, expensive, and dangerous [11,12].

Pharmaceutical companies that produce drugs in ampoules for injection recommend intravenous injections more often than intramuscular injections, and subcutaneous injections are contraindicated for most drugs [[13], [14], [15]].

Despite their significant danger, intravenous injections have long been widely used for administration of drugs. For a long time, no one noticed or explained this paradox. For decades, medical professionals worldwide have followed the Guidelines for Injection Drug Use in hospitals without addressing intravenous injection safety [16]. However, more recently, people with drug addictions and patients with cancer have realized the dangers of intravenous injections. Because these people receive multiple intravenous opioid injections, they are more likely to develop localized post-injection complications. Iatrogenic post-injection abscess affect many people with drug addictions and patients with chronic pain. Some physicians have tried to reduce opioids and other intravenous injections after witnessing this unintentionally. [17,18]. However, intramuscular, subcutaneous, and other drug injections do not prevent general or local complications [[19], [20], [21]].

The proportions of clinicians using intravenous, intramuscular, and subcutaneous injections of drugs showed that neither compliance with all rules of these injections nor use of high-quality drugs for injections could preclude the development of general and local post-injection complications, as seen after injections of steroids and nonsteroidal anti-inflammatory drugs [[22], [23], [24], [25]].

Among complex local post-injection complications, reversible and irreversible local inflammation, severe local pain syndrome, necrosis, and abscess are the most unpredictable and unexplained [[26], [27], [28]]. This specific complication was described approximately 100 years ago [29], and is now called Nicolau syndrome [[30], [31], [32], [33], [34], [35]]. These complications are not caused by injection of any specific drug, and cannot be explained by the known mechanisms of drug action. Additionally, Nicolau syndrome can develop after injections of local anesthetics, steroids, nonsteroidal anti-inflammatory drugs, chemotherapeutic agents, vaccines, and a combination of drugs [20,[36], [37], [38], [39]]. Tachon syndrome, similar to Nicolau syndrome, describes post-injection complications of steroid injection into an artery and vein [[40], [41], [42]].

The dominant view among researchers is that these local complications arise from violations of injection technology, primarily the rules of asepsis and antisepsis [43], as well as infection [36,44,45]. However, these notions do not explain cases of instant local pain, inflammation, necrosis, or abscesses at the drug injection site [45,46]. Additionally, post-injection complications are often aseptic [7,24,27,28,43].

In recent years, the drugs in the injection solution have been seen to induce localized post-injection complications (up to and including abscesses) [47,48]. High-quality drugs can cause local inflammation, necrosis, and abscesses at injection sites [49,50]. Moreover, the current injection solution quality control standard does not assess post-injection pain syndrome, inflammation, necrosis, or abscesses [51]. Drug irritation can be assessed by local tissue inflammation at the injection sites, and inflammation can be assessed by local temperature dynamics. Modern thermal imaging cameras and their use in biology and medicine allow infrared thermography to detect local inflammation at injection sites in real time [[52], [53], [54], [55], [56]].

This study aimed to investigate the possibility of using dynamic thermography to assess the quality and safety of drugs and injections.

## 3 Methods

Ten healthy 2-month-old Landrace piglets were used to study abdominal skin temperature dynamics before and 5 min after injecting 0.5 mL solutions of 20 high-quality drugs.

Osmotic activity of the drug solutions was determined using the cryoscopic method with an osmometer (OSMO-MAT-030 RS, ANSELMA Industries, Austria). This technique is based on registering the temperature regime of the solution under study at which crystallization occurs. The acid activity of the drug solutions was determined by the potentiometric method for analyzing pH, using universal ionometers authorized by the Pharmacopoeia.

On five healthy adult volunteers, the dynamics of local temperature in the skin of the shoulder or gluteal muscle was studied during the first 5 min after subcutaneous injection of 0.5 mL of 0.9 % isotonic sodium chloride solution or 5 % glucose solution. The study protocol on humans and experimental animals (piglets) was conducted in accordance with the Declaration of Helsinki [57] and was approved by the Ethics Committees at the Izhevsk State Medical Academy (Protocol No. 477, April 16, 2016) and the Institute of Thermology (Protocol No. 1 of September 27, 2016).

Before starting the study, all the volunteers provided written informed consent. To study the dynamics of the local skin temperature at the injection site, 0.5 mL of 0.9 % sodium chloride solution and 5 % glucose solution were used at room temperature, as is customary in clinical practice (temperature of 24–26 °C). The solutions were injected into the skin using needles designed for subcutaneous injections.

Skin changes were documented using photographs, and skin temperature was recorded using a Thermo Tracer TH9100XX thermal imager (NEC, USA). The ambient temperature in the examination room was 24 ± 0.5 °C, and the temperature window of the thermal imaging camera was set in the range of 25–36 °C [58,59]. Infrared thermal images of the skin at the injection sites were recorded before, during, and after injections at 30 s intervals. The area of interest was determined throughout the body part under study using its contour to separate its temperature from the background. The obtained data were processed using the Thermography Explorer and Image Processor software. Quantitative data are presented as arithmetic means (M) and standard deviations (SD).

## 4 Results

We evaluated the dynamics of local temperature in the skin of the anterior abdominal wall in piglets after injecting 0.5 mL solutions of several drugs. Before this, we investigated the osmotic activity and pH of the drug solutions. This was performed to divide the drugs into groups based on the magnitude of osmotic activity and acidity. In the first group of drugs, we included drugs with an osmotic activity of <280 mOsmol/L of water and acidity with pH values between 7.0 and 4.35. Table 1 shows the results.

**Table 1:** The value of osmotic activity and acidity (alkalinity) of drug solutions (group 1).

Drugs	Mosmol/L water	pH
Water for injection	5.1 ± 0.3	5.22 ± 0.05
0.9 % Sodium chloride solution	278 ± 5.0	6.15 ± 0.05
1 % Novocaine solution	78.6 ± 1.6	4.35 ± 0.04
2 % Lidocaine hydrochloride solution	167 ± 4.6	5.85 ± 0.06
1 % Cefazoline sodium salt solution	52 ± 1.7	5.61 ± 0.06
1 % Benzylpenicillin sodium salt solution	49.1 ± 1.9	5.77 ± 0.06
Results (SD & Average)	104.96 ± 100.38 (5.1–278)	5.49 ± 0.64 (4.35–6.15)

In the second group of drugs, we included drugs with osmotic activity of >1500 mosmol/L of water and acidity with pH values between 8.5 and 5.9. Table 2 shows the results.

**Table 2:** The value of osmotic activity and acidity (alkalinity) of drug solutions (group 2).

Drugs	Mosmol/L water	pH
Solution of 10 % sodium chloride	2951 ± 5.5	6.22 ± 0.1
Solution of 10 % lidocaine hydrochloride	796 ± 9.5	5.85 ± 0.06
Solution of 10 % calcium chloride	1293 ± 7	5.9 ± 0.08
Solution of 20 % sodium sulfacyl	1618 ± 15.3	8.2 ± 0.09
Solution of 50 % sodium metamizole	3300 ± 12	6.65 ± 0.08
Solution of 76 % urografin	3900 ± 5	6.9 ± 0.1
Results (SD & Average)	2309.66 ± 1242.93 (796–3900)	6.62 ± 0.87 (2.95–8.0)

In the third group of drugs, we included hypotonic or isotonic drug solutions with pH values between 2.95 and 6.0. Table 3 shows the results.

**Table 3:** The value of osmotic activity and acidity (alkalinity) of drug solutions (group 3).

Drugs	Mosmol/L water	pH
5 % Ascorbic acid solution	540 ± 5.0	6.0 ± 0.1
5 % Pyridoxine solution	404 ± 4.5	2.95 ± 0.06
1 % Nicotinic acid solution	139 ± 2.0	6.0 ± 0.08
0.5 % Cavinton solution	826 ± 7.3	3.2 ± 0.09
5 % Glucose solution	305 ± 1.6	3.7 ± 0.1
5 % Thiamine chloride solution	362 ± 1.5	3.0 ± 0.1
Results (SD & Average)	429.3 ± 234.31 (139.0–826.0)	4.14 ± 1.463 (2.95–6)

The third group of drugs included diclofenac and ortho-phenol (24 % alcohol solution). The results are summarized in Table 4.

**Table 4:** The value of osmotic activity and acidity (alkalinity) of drug solutions (group 4).

Drugs	Mosmol/L water	pH
Diclofenac	3695 ± 9.0	8.3 ± 0.1
Ortophen	3952 ± 13.1	6.2 ± 0.05
Results (Standard deviation and Average)	3823.5 ± 181.726 (3695.0–3952.0)	7.25 ± 1.485 (6.2–8.3)

The results showed that the solutions of all the drugs had different osmotic and acidic activities. We compared the data obtained with the Pharmacopoeia requirements for the quality of the drugs studied. The comparison result showed that the detected acidity (alkalinity) values corresponded to the permissible limits of the values, and the osmotic activity of the drugs was uncontrolled.

Subsequently, the dynamics of the local temperature of piglet skin at the injection site of the drug solutions were studied, considering their osmotic and acidic activities, with the results showing their high diagnostic and prognostic value. These dynamics depend on the degree of difference between their osmotic and acidic activities from physiological values, reflecting the intensity of post-injection local inflammation and indicating the likelihood of developing necrosis and abscess a few hours and days after injection.

Therefore, in the first series of experiments, subcutaneous injections of drugs with osmotic activity of <280 mosmol/L of water and acidity not lower than pH 4.35, the skin temperature at the injection site initially decreased slightly, after which it rose to normal, and then slowly and increased by ≤ 1 °C compared to the initial value. Local hyperthermia was combined with local hyperemia, which was short-term and reversible, and no abscesses formed after drug injection.

In the second series of experiments with subcutaneous injections of drugs with immensely high hyper-tonic activity and with insignificant acidic or alkaline activity, skin temperature at the injection site initially decreased slightly; after 5 min, it increased by >3 °C compared with the initial value. Local hyperthermia was accompanied by an erythematous spot on the skin at the injection site, and after 2–3 days, a post-injection abscess formed.

In the third series of experiments, subcutaneous injections of 0.5 mL of drugs with a pH of 2.95–6.0 and an osmotic activity of less than 280 mosmol/L of water reduced the local temperature in the 1st minute. The temperature in the skin increased, but >2°С and remained up to 12 h after injection. However, an erythematous spot on the skin, post-injection necrosis, and abscess did not form.

In the fourth series of experiments, the local skin temperature at the injection site gradually increased by 3.5 °C 5 min after injection of diclofenac or orthophen (0.5 mL) (see Table 5). At the same time, a bright red erythematous spot formed on the skin, and after 2 days, a post-injection abscess formed.

**Table 5:** Mean values of temperature in the area of injection in piglets.

	T_0_	T_30_	T_60_	T_180_	T_300_
0,9% sodium chloride solution	30.7 ± 0.908 (29.6–32.3)	27.91 ± 1.009 (26.4–29.1)	29.3 ± 1.331 (26.9–31.3)	29.28 ± 1.027 (27.9–31.1)	30.07 ± 1.346 (28.4–32.1)
50 % sodium metamizole solution	30.7 ± 0.908 (29.6–32.3)	29.56 ± 1.152 (27.6–31.5)	29.05 ± 1.274 (27.8–31.8)	31.2 ± 1.072 (29.0–32.5)	32.08 ± 1.746 (29,. −34.6)
5 % glucose solution	30.7 ± 0.908 (29.6–32.3)	290.1 ± 2.440 (26.3–33.6)	29.11 ± 1.415 (279.-32.7)	29.56 ± 1.259 (27.9–31.5)	31.33 ± 1.530 (29.1–33.1)
Orthophene	30.7 ± 0.908 (29.6–32.3)	29.4 ± 0.923 (27.9–31.0)	29.84 ± 1.377 (28.5–32.8)	30.17 ± 1.343 (28.0–32.1)	31.71 ± 1.266 (29.6–33.2)
10 % metamizole sodium solution	30.7 ± 0.908 (29.6–32.3)	28.99 ± 0.647 (27.9–30.2)	29.09 ± 1.104 (27.3–30.8)	29.57 ± 1.354 (27.9–31.8)	31.14 ± 0.935 (29.6–32.3)

In the fifth series of experiments, the dynamics of local skin temperature at the injection sites of drugs included in groups 2 and 4 were studied after preliminary dilution five times with water for injection. The results showed that injections of diluted drugs caused local hyperthermia, hyperemia, and skin infiltration at the injection site without the formation of erythematous spots on the skin or post-injection abscesses.

**Fig. 1:**
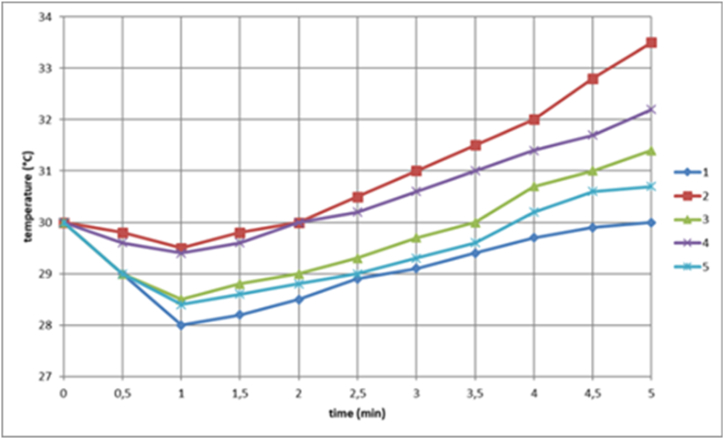
The dynamics of the local temperature of the piglet's skin at the injection site of “reference” (indicative) drugs, which is a qualitative and quantitative thermogram of drugs from 4 groups of drugs (1: 0.9 % sodium chloride solution; 2: 50 % sodium metamizole solution; 3: 5 % glucose solution; 4: orthophen; 5: a solution of 10 % metamizole sodium).

Fig. 1 shows an indicative diagram reflecting the dynamics of the local skin temperature at the injection site of the “reference” (indicative) medicine from each of their four groups of drugs.

From the above illustration, the local temperature in the skin at the injection site is clearly observed to decrease in the first minute after drug injection. This is caused by the cooling of the skin, since the drug solutions were at room temperature (24–26 °C). Subsequently, we investigated the local temperature of piglets at the injection site after injection of a certain drug. The mean values with standard deviations were analyzed and calculated, and the data obtained are summarized in Table 2. Subsequently, the skin temperature increased, but the intensity of temperature increase and the magnitude of hyperthermia were different. In the subsequent development of an erythematous spot in the skin and a post-injection abscess, the local temperature in the skin increased very intensively and, 5 min after injection, exceeded the initial temperature by more than 2°. Conversely, Table 6 shows that in cases where there was no formation of erythematous spots in the skin and post-injection abscesses, the local temperature in the skin at the injection site increased by no more than 2°.

**Table 6:** Temperature difference in the injection area, where ΔT30 = T0-T30, ΔT60 = T0-T60, ΔT180 = T0-T180, ΔT300 = T0-T300.

	ΔТ30	ΔТ60	ΔТ180	ΔТ300
0,9 % sodium chloride solution	−2.79	−1.4	−1.42	−0.63
50 % sodium metamizole solution	−1.14	−1.65	0.5	1.38
5 % glucose solution	−1.69	−1.59	−1.14	0.63
Orthophene	−1.3	−0.86	−0.53	0.01
10 % metamizole sodium solution	−1.71	−1.61	−1.13	0.47

**Fig. 2:**
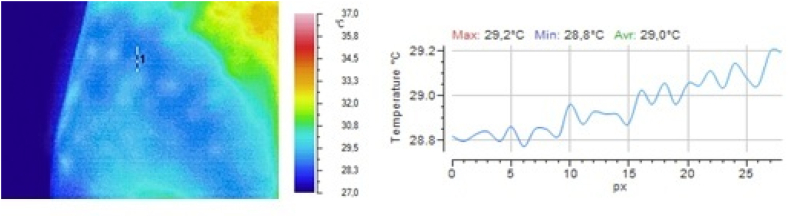
Local temperature of the selected gluteal part of the volunteer before injecting the drug.

**Fig. 3:**
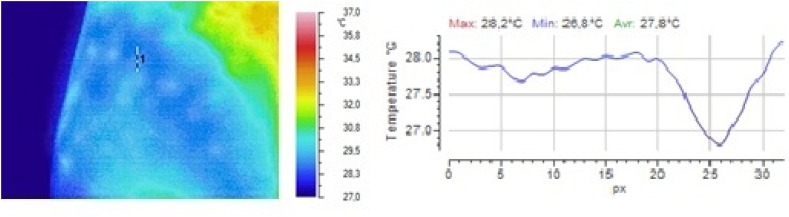
Local temperature of the selected gluteal part of the volunteer 1 min after subcutaneous injection of 0.5 ml of 0.9 % sodium chloride solution.

**Fig. 4:**
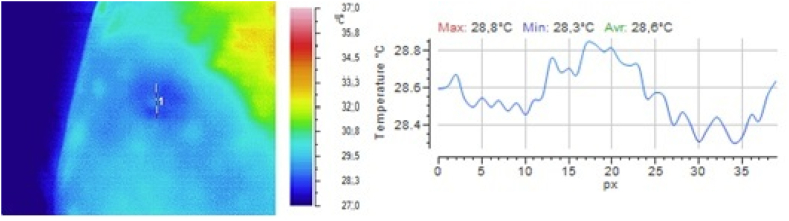
Local temperature of the selected gluteal part of the volunteer 5 min after subcutaneous injection of 0.5 ml of 0.9 % sodium chloride solution.

**Fig. 5:**
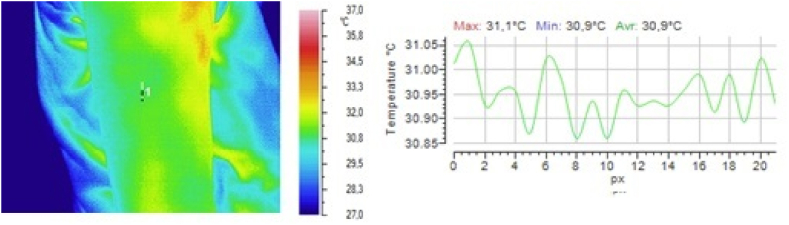
Local temperature of the selected part of the volunteer's shoulder before administration of the drug.

**Fig. 6:**
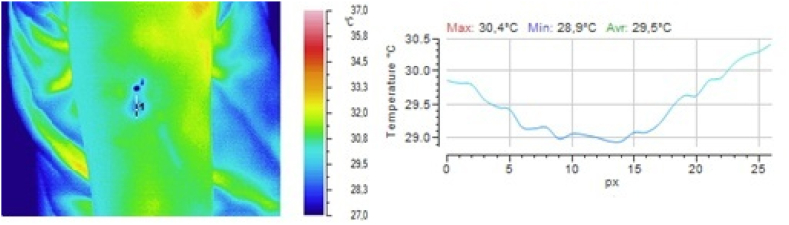
Local temperature of the selected part of the volunteer's shoulder 1 min after subcutaneous injection of 0.5 ml of 5 % glucose solution.

**Fig. 7:**
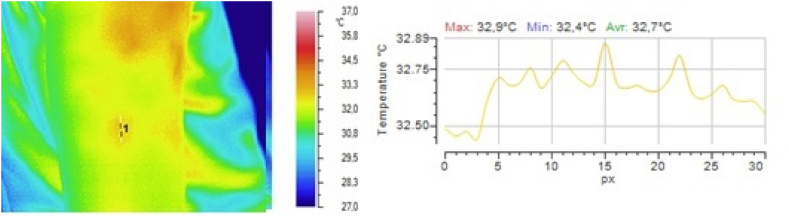
Local temperature of the selected part of the volunteer's shoulder 5 min after subcutaneous injection of 0.5 ml of 5 % glucose solution.

In one group of volunteers, the dynamics of the local temperature in the skin at the subcutaneous injection site from group 1) and a 5 % glucose solution (a reference drug from group 3) were studied. Drugs from groups 2 and 4 were not injected because of their low safety. The results obtained after injection of 0.9 % sodium chloride solution are shown in Fig. 2, Fig. 3, Fig. 4, and those obtained after injection of 5 % glucose solution are shown in Fig. 5, Fig. 6, Fig. 7.

It is clear from the above illustrations that infrared thermography provides real-time visualization of the foci of local hypothermia and local hyperthermia in the skin at the injection site of drugs. Furthermore, in the first 5 min after injection, infrared monitoring was seen to allow detection of the focus of local hyperthermia and quantitative determination of temperature values in this focus.

## 5 Discussion

Globally, drugs in the dosage form “solution for injection” continue be the most frequently used mode of drug administration used in treating various diseases. High-quality intravenous and intramuscular injections induce local post-injection complications, including local pain syndrome, aseptic inflammation, necrosis, and abscess [[60], [61], [62]]. These symptoms cause Nicolau syndrome, an iatrogenic condition [63,64]. The etiology, pathogenesis, and prevention of iatrogenic diseases are poorly understood [27,65]. Because the current drug quality control standard does not evaluate local irritation and inflammatory effects on tissue at injection sites, any drug produced with these properties unknowingly by a drug manufacturer (pharmaceutical company) may cause aseptic inflammation, necrosis, and abscess after injection [19,20]. Some drug solutions may have very high concentrations of ingredients, making them hypertonic, and a significant proportion may have extremely low pH values, making them acidic [66]. Moreover, some injection solutions may contain alcohols or aldehydes in cauterizing concentrations [20,26].

The current standard for drug quality control does not provide a real-time assessment of the temperature dynamics of injection sites [51]. Under these conditions, drugs may be hypertonic, acidic, or alcoholic solutions, which may cause local irritating and cauterizing effects on soft tissue during intradermal, subcutaneous, intramuscular, intravenous, and other injections. Pharmacopoeia specifies the relevant requirements for the quality and safety of drugs, but allows drugs in the dosage form “Solution for injection” to have pH values < 7.4 or an osmotic activity value > 300 mosmol/L water, and to contain alcohols, aldehydes, phenols or heavy metal salts [19,20,51]. These local complications after drug injection are unrelated to their cauterizing effect [67]. However, mid-20th century reports suggested that hypertonic sodium chloride injections can cause aseptic necrosis [68], and several studies have confirmed this [69,70]. Nicolau syndrome can also result from hypertonic calcium chloride, calcium gluconate, or calcium hydroxide injections [20,71]. However, most researchers have not reported these findings.

In our study, we assumed that uncontrolled local irritating or cauterizing (necrotizing) activity of the injection solutions facilitated the development of post-injection aseptic inflammation, necrosis, and abscess [19,20]. Therefore, monitoring the local skin temperature at the injection site is recommended to assess nonspecific (physical-chemical) aggressive activity of drugs. We relied on reports that recounted development of local inflammation, infiltration, and tissue soreness within the first 2 min after injection [7,27]. These data were consistent with the results of sonographic monitoring of tissues at the injection site during NAS development of Nicolau syndrome [72]. Moreover, monitoring damaged skin has been reported to allow detection of malignant skin tumors and foci of local aseptic inflammation, such as in an additional breast lobe [[73], [74]]. The possibility of using infrared to diagnose local post-injection complications through visualization of the zone of local hyperthermia in the skin at the injection site was first presented at the beginning of the 21st century [[75], [76], [77]].

Furthermore, we assumed that infrared monitoring of local skin temperature at the site of subcutaneous drug administration was the only way to assess its local irritating and cauterizing effects. However, the skin and subcutaneous fat are particularly susceptible to the local irritating and cauterizing effects of drugs during injections. The skin illustrates Nicolau syndrome development for a reason [7,27,28]. The prohibition on subcutaneous injections of many drugs is likely due to the high sensitivity of the skin and subcutaneous fat to drug irritation. Due to the lack of control over the dynamics of local skin temperature at injection sites, drugs may cauterize skin and subcutaneous fat. Two-month-old Landrace piglets were used for the first experiments because their drug sensitivity was considered to be similar to that of humans. Pigs and humans have similar skin anatomy, and physiology, and piglets are more reactive than adult pigs. The results of the experiments supported our hypotheses. Furthermore, the results of experimental studies have shown that infrared monitoring of skin temperature at the injection site within 5 min after drug administration makes it possible to assess the local irritant activity of a given drug based on its osmotic activity, acid activity, or the presence of alcohols and aldehydes at a cauterizing concentration.

Thus, in the first series of experiments, subcutaneous injections of 0.5 mL of mildly acidic hypotonic or isotonic preparations, with no alcohols or aldehydes in a cauterizing concentration, caused the following at the injection site: a slight decrease in skin temperature, followed by a return to normal, followed by a slow and slight increase of <1 °C 5 min after injection. Simultaneously, local hyperthermia was associated with local hyperemia and skin infiltration. These symptoms of inflammation persisted 1–1.5 h before disappearing. No post-injection abscesses were found. Post-injection inflammation was short-term and reversible.

In the second series of experiments, subcutaneous injections of 0.5 mL of hypertonic solutions of drugs did not show excessive acid or alkaline activity, because their osmotic activity exceeded 1500 mosmol/L of water. These solutions fell in the pH range of 8.5–5.9, and caused the following dynamics of local skin temperature at the injection site: immediately after injection, skin temperature decreased slightly, before gradually increasing. At the same time, 1, 2, and 5 min after injection, the temperature exceeded the initial values by 0.2–0.5 °C, 1.0–2.0 °C, and 2.5–4.0 °C (respectively). After injecting all drugs except urografin, the skin and subcutaneous fat showed an infiltrative zone and vivid erythema in the skin. The most intense and pronounced local hyperthermia and erythematous skin coloration at the injection site were detected after administering 10 % calcium chloride solution. After injection of 76 % urografin solution, a white-gray area formed in the skin at the injection site. Post-injection abscesses were formed 3 days after injection of solutions of 10 % sodium chloride, 10 % calcium chloride, 50 % metamizole sodium, and 76 % urografin. Consequently, these drugs (or hypertensive medicinal solutions) cause acute post-injection inflammation that quickly becomes irreversible. All drugs were hypertensive solutions. They were antiseptic, so they could not cause infection. Therefore, post-injection inflammation and abscesses caused by them were aseptic. In our opinion, intradermal erythema, which develops at the site of local post-injection inflammation and rapidly becomes irreversible, is also caused by an overly strong nonspecific local effect of drugs (i.e., the aggressive physicochemical action of a hypertonic solution, causing damage to the walls of blood vessels and blood spilled from them into the skin tissue).

In the third series of experiments, subcutaneous injections of 0.5 mL of hypotonic or isotonic solutions of excessively acidic drugs caused the following dynamics of local skin temperature at the injection site: immediately after injection, skin temperature decreased slightly, then rose to the initial level, and 2 and 5 min after injection, exceeded the initial values by 0.2–0.5 and 1.0–2.0 °C (respectively). Simultaneously, local infiltration and hyperemia developed in the skin at the injection site. After 12 h, all symptoms of local inflammation disappeared. Post-injection necrosis and abscesses were absent.

In the fourth series of experiments, 0.5 mL of diclofenac or orthophen injection (24 % alcohol solution), increased local skin temperature by 0.2–0.4 °C, 0.8–1.8 °C, and 2.3–3.5 °C (respectively), 1, 2, and 5 min after injection. After the injection of these drugs, an infiltration zone was seen in the skin and subcutaneous fat at the injection site, and a bright red erythematous spot was found. Visually and sonographically, inflammation and infiltrates were seen, which transformed into post-injection abscesses after 2 days.

In the fifth series of experiments on piglets, the dynamics of the local skin temperature at the site of subcutaneous injection (0.5 mL) of each solution from the group of drugs that caused irreversible post-injection inflammation and post-injection abscesses in **experiments 2 and 4** were studied. However, no significant difference was observed; each drug was diluted with water for injection five times before injection. Subcutaneous injections of diluted drug solutions caused short-term local hyperthermia, hyperemia, and skin infiltration at the injection site without formation of erythematous spots or post-injection abscesses.

Studies on healthy adult volunteers were conducted on the dynamics of the local temperature in the skin at the injection sites of safe and conditionally safe drugs, namely, a solution of 0.9 % sodium chloride and a solution of 5 % glucose (pH 3.7). The results showed that the dynamics of local skin temperature detected by infrared thermography at drug injection sites allowed real-time evaluation of the local irritant effect and prediction of the outcome of a local drug interaction.

In all experimental animals, subcutaneous injections of hypertonic or hyper-acidic drug solutions rapidly raised skin temperature by several degrees. Hypertonic drug solutions with cauterizing action can increase the local skin temperature at the injection site by > 3 °C 5 min after subcutaneous injection. Injections of the same solutions, but diluted five times with water, increased the local skin temperature slightly 5 min after the start of the local interaction. It is crucial to note that the preliminary dilution of cauterizing solutions with water eliminates their post-injection hyperthermic effect and completely prevents the development of post-injection complications, such as post-injection erythema of the skin and post-injection abscesses.

The results showed that infrared monitoring of piglet skin temperature at drug injection sites allowed for real-time assessment of the intensity and direction of changes in the local temperature. In particular, post-injection hyperthermia, which develops intensively and reaches high values 5 min after injection, indicates a high probability of local post-injection inflammation of an irreversible nature, necrosis, and post-injection abscess. The developed thermal imaging technology and methods are easily applicable in clinical practice, although an automated approach would likely be preferred for its implementation. In particular, the experience of machine learning obtained from using thermal imaging to assess vascular diseases in clinical practice can be used for this purpose [78].

However, further research is required to confirm these hypotheses. Such studies may supplement the informativeness of the developed method in the future.

## 6 Conclusion

Drug and injection technology safety can be assessed by measuring local skin temperature for 5 min at the injection site. The absence of local hyperthermia of the skin at the injection site within 5 min after injection indicates that drugs do not cauterize or irritate the skin, and may indicate that the drug is safe for tissues at this site. A zone of local hyperthermia in the skin at the injection site in the 1st 2 min after injection, along with a progressive increase in local temperature for at least 5 min, indicate a strong local irritating or cauterizing effect of the drug as well as a high probability of developing aseptic post-injection inflammation, infiltration, necrosis, and abscesses (Nicolau syndrome). Thus, thermal monitoring of local skin temperature for 5 min after subcutaneous or intradermal injection of a drug can be considered an integral part of the quality and safety assessment of drugs and injections.

## Ethics statement

The protocol of the study on humans and experimental animals (piglets) corresponded to the principles set out in the Helsinki Declaration of the World Health Organization, and was approved by the Ethics Committees at the Izhevsk State Medical Academy (Protocol No. 477, April 16, 2016) and at the Institute of Thermology (Protocol No. 1 of September 27, 2016).

## Data availability statement

The data that support the finding of this study are available from the corresponding author upon reasonable request.

## Patents

1. Method for testing the quality of medicinal preparations. RU Patent No. 2304769.
2. Method for testimating local drug toxicity. RU Patent No. 2396562.
3. Method for monitoring irritant effect of intravascular catheters. RU Patent No. 2405585.
4. Method for infrared phlebography. RU Patent No. 2638458.

## CRediT authorship contribution statement

**Aleksandr Urakov:** Writing – review & editing, Methodology, Data curation, Conceptualization. **Natalya Urakova:** Writing – original draft, Data curation, Conceptualization. **Aleksandr Samorodov:** Validation, Resources, Formal analysis. **Petr Shabanov:** Supervision, Methodology, Conceptualization. **Ilnur Yagudin:** Methodology, Formal analysis. **Anastasia Stolyarenko:** Writing – review & editing, Writing – original draft, Validation, Software. **Darya Suntsova:** Writing – original draft, Methodology, Formal analysis. **Nikita Muhutdinov:** Writing – original draft, Methodology, Formal analysis.

## Declaration of competing interest

The authors declare that they have no known competing financial interests or personal relationships that could have appeared to influence the work reported in this paper.
